# Anorectal *Chlamydia trachomatis* Load Is Similar in Men Who Have Sex with Men and Women Reporting Anal Sex

**DOI:** 10.1371/journal.pone.0134991

**Published:** 2015-08-11

**Authors:** Geneviève A. F. S. van Liere, Jeanne A. M. C. Dirks, Christian J. P. A. Hoebe, Petra F. Wolffs, Nicole H. T. M. Dukers-Muijrers

**Affiliations:** 1 Department of Sexual Health, Infectious Diseases and Environmental Health, South Limburg Public Health Service, Geleen, the Netherlands; 2 Department of Medical Microbiology, School of Public Health and Primary Care (CAPHRI), Maastricht University Medical Center + (MUMC+), Maastricht, the Netherlands; University of California, San Francisco, University of California, Berkeley, and the Children's Hospital Oakland Research Institute, UNITED STATES

## Abstract

**Background:**

Anorectal *Chlamydia trachomatis* (chlamydia) is frequently diagnosed in men who have sex with men (MSM) and in women, but it is unknown whether these infections are comparable in clinical impact and transmission potential. Quantifying bacterial load and identifying determinants associated with high bacterial load could provide more insight.

**Methods:**

We selected a convenience sample of MSM who reported anal sex (n = 90) and women with concurrent urogenital/anorectal chlamydia who reported anal sex (n = 51) or did not report anal sex (n = 61) from the South Limburg Public Health Service’s STI unit. Bacterial load (Chlamydia/ml) was quantified for all samples and log transformed for analyses. Samples with an unquantifiable human leukocyte antigen (n = 9) were excluded from analyses, as they were deemed inadequately sampled.

**Results:**

The mean log anorectal chlamydia load (3.50) was similar for MSM and women who reported having anal sex (3.80, P = 0.21). The anorectal chlamydia load was significantly higher in these groups than in women who did not report having anal sex (2.76, P = 0.001). Detectable load values ranged from 1.81–6.32 chlamydia/ml for MSM, 1.74–7.33 chlamydia/ml for women who reported having anal sex and 1.84–6.31 chlamydia/ml for women who did not report having anal sex. Symptoms and several other determinants were not associated with anorectal chlamydia load.

**Conclusions:**

Women who did not report anal sex had lower anorectal loads, but they were within a similar range to the other two groups. Anorectal chlamydia load was comparable between MSM and women who reported anal sex, suggesting similar transmission potential.

## Introduction

Anorectal *Chlamydia trachomatis* (chlamydia) is frequently diagnosed in sexually transmitted infection (STI) clinics. International guidelines advise using nucleic acid amplification tests (NAAT) to test anorectal specimens because they are more sensitive than the use of culture [1,2]. Moreover, self-collected rectal samples are as feasible, valid, and acceptable for testing as provider-collected swabs [3,4]. Anorectal testing is mainly indicated in risk groups such as men who have sex with men (MSM); in some countries, guidelines also include women at high risk such as prostitutes, swingers and those with multiple sex partners. Testing is generally guided by report of receptive anal sex and/or anal symptoms [5,6].

Anorectal infections detected by a NAAT in MSM are generally assumed to be real infections and are given adequate treatment to halt transmission and reduce complications in individuals. Additionally, in recent years, several published studies have recommended anorectal testing for women because of the substantial prevalence of anorectal chlamydia among them (6–15%) [7–9]. The prevalence of anorectal chlamydia in women is strikingly similar to that in MSM (7–14%) [10–12]. Women do frequently report anal sex (11–26%) as do MSM [9,13–15]. Nonetheless, recent studies using routine anorectal testing have found that over half to two-thirds of anorectal chlamydia infections were observed in women and MSM who did not report anal sex [9,16,17]. This finding fuels international debate about who to test for anorectal chlamydia infections.

Although the prevalence is similar between MSM and women, anorectal chlamydia infections in women are mainly concurrent with urogenital infections, in contrast to MSM [8–10,12,18,19]. This may give rise to questioning whether anorectal infections in MSM and women detected by NAAT are comparable in clinical impact and transmission potential. The clinical impact may be considered in terms of symptoms. The majority of anorectal chlamydia infections in MSM and women are asymptomatic. Transmission potential could be carefully considered in terms of bacterial load (e.g. high loads indicate a higher transmission potential for viral STIs) [20]. Therefore, targeting individuals with high chlamydia loads could have an added value in practice.

To date, only a few studies have used a NAAT to report on anorectal load [15,21,22], and none of these studies have compared anorectal loads between MSM and women. Yet, quantifying bacterial load and knowing which determinants are associated with high bacterial load could provide a unique insight into possible clinical (i.e. symptoms) and public health (i.e. transmission) aspects of anorectal infections. Additionally, comparing bacterial load and associated determinants between MSM and women could help to ascertain whether their anorectal infections are equally severe in terms of symptoms and transmission. Moreover, anorectal load could differ between women who report anal sex and women who do not report anal sex, due to different transmission routes (i.e. autoinoculation from vaginal secretions in women who do not report anal sex). This study quantified bacterial load in chlamydia positive anorectal samples from MSM who reported anal sex (tested as recommended in STI guidelines [5]) and from women with concurrent urogenital chlamydia, and assessed determinants associated with bacterial load.

## Methods

### Study population and procedures

The South Limburg Public Health Service’s STI unit provides more than 6000 consults annually, offering free examination and treatment at three regional outpatient STI clinics. Clients are routinely tested at urogenital sites for *Chlamydia trachomatis* and *Neisseria gonorrhoeae*. Men who report having had sex with men in the past six months are also routinely tested anorectally. Until May 2012, women were anorectally tested based on self-report of anorectal symptoms and/or anal sex. From May 2012, all women were routinely tested anorectally.

Specimens were self-collected vaginal swabs, anorectal swabs and urine. Trained STI nurses asked patients to take a self-collected anorectal swab and provided them with verbal instructions and a diagram (i.e. insert the swab 2.5 cm into the rectum, rotate for 5–10 seconds, and place the swab in the capped tube). Specimens were processed at the department of Medical Microbiology at Maastricht University Medical Center + (Maastricht, The Netherlands) using nucleic acid amplification tests (NAAT) [polymerase chain reaction PCR; Cobas Amplicor until 2012 and afterward Cobas 4800, both from Roche Diagnostics, San Francisco, CA]. Serum was tested for *Treponema pallidum* hemagglutination antigen (TPHA) and human immunodeficiency virus (HIV) (anti-HIV[1/2], Axsym; Abbott Laboratories, Chicago, IL). Reactive samples were confirmed using Western blot (HIVblot 2.2; Genelabs Diagnostics, Science Park, Singapore), according to the manufacturer’s protocol. In addition to testing, trained study nurses took a standardised medical and sexual history at each consult, which included demographic data, self-reported symptoms, sexual behaviour in the preceding six months and antibiotic use in the past month. Anal symptoms (proctitis) included rectal discharge, bleeding, pain, redness, burning sensation or itching. All data were registered in an electronic patient registry.

### Study selection

Between November 2010 and September 2013, 796 anorectal chlamydia infections were diagnosed in MSM who reported anal sex and in women with concurrent urogenital chlamydia. We selected a convenience sample of MSM who reported anal sex and women with concurrent urogenital chlamydia who were 16 years or older and positive for non-LGV anorectal chlamydia for this study; this yielded 211 consults by 194 individuals for further analyses. The study sample (26.5% from total sample) was selected based on availability and easy access. To assess whether selection bias had occurred in the sample, we used a Chi-square test to compare age, nationality and sex between persons who were included in the study and MSM reporting anal sex and women who tested positive for anorectal chlamydia who were not included in the study. Individuals in the study sample were on average younger than those not included (mean 30 years (inter quartile range (IQR) 22–44) versus 34 years (IQR 21–41), P<0.001). The proportion of individuals with a non-Western nationality was comparable between individuals included and not included (4.8% n = 10 versus 3.8% n = 19, P = 0.56). The proportion MSM included was smaller compared to women (19.6% n = 96, versus 37.5% n = 116, P<0.0001).

This study was approved by the Medical Ethical Committee of the University of Maastricht (METC 11-4-108), who waived the need for consent to be collected from participants. Since retrospective data originated from standard care (in which one can opt-out for the use of their data for scientific research, as approved by METC 11-4-108) and were analyzed anonymously, no further informed consent for data analysis was obtained.

### Nucleic acid extraction and quantitative CT polymerase chain reaction (PCR)

Total nucleic acids from each 200μl sample were isolated using the QIAamp DNA Mini kit (Qiagen, Hilden, Germany) according to the manufacturer’s instructions and eluted in 120 μl. The eluate was stored at -20°C and thawed once for quantification. Prior to DNA-isolation, an internal extraction and amplification control and a negative extraction control were added to each sample, as described elsewhere [23,24].

Quantitative PCR for CT used primers targeting the single-copy *OmpA* gene, coding for the major outer membrane protein, as described by Jalal et al. [25]. For eukaryotic cell determination, primers targeting the MHC class II antigen (HLA-DQA1) gene were used, as described by van der Helm et al. [26], as a test for adequacy of the sampling. For absolute quantification, the *ompA* and HLA-DQA1 PCR products were cloned separately into the pGEM-T easy vector (Promega Corporation, Madison, WI, USA) according to the manufacturer’s protocol. Plasmids were isolated using alkaline lysis and purified using phenol/chloroform extraction, as described previously [27]. Logarithmic dilutions covering a 5-log range were made (converted to correspond with 10^6^ to 10^2^ copies/ml in clinical samples).

QPCR was performed with a 7900HT Real-Time PCR System (Applied Biosystems, Foster City, California). In each run, the 96-micro-wells plate contained both dilution series, a negative control and the samples for quantification.

PCR amplification was performed in a total volume of 25 μl, consisting of a 10 μl template and a 15 μl reaction mixture containing 12.5 μl Absolute qPCR Rox Mastermix (Thermo Scientific, Waltham, USA) and a 2.5 μl primer/probe mix consisting of 840 nM forward and reverse primer and 100 nM probe. The amplification reaction consisted of 15 minutes of initial activation at 95°C, followed by 42 cycles at 95°C for 15 seconds and 60°C for 60 seconds.

### Load determination

Absolute chlamydia loads (log chlamydia/ml) were obtained by entering cycle threshold (Ct) values into a master curve (compiled from over 10 dilution series), and then exponentially transformed. Samples were deemed inadequately sampled and excluded from further analysis when no human cells (HLA-DQA1) were detected or no target could be detected at all; this resulted in excluding 9 of the 211 samples. The initial diagnostic chlamydia screening was done with assays targeting the chlamydial plasmid, which are both present in relative abundance to the single-copy *OmpA-* gene used in our load assessment.

For this reason, some samples contained loads below the quantification range of the *ompA* PCR. These ‘low load’ samples, which were proven positive at the initial screening, were set to a Ct value >42 which resulted in a load of 0.75 log chlamydia/ml[28].

### Statistical analyses

Load values were log transformed for analyses. The study sample consisted of three non-overlapping indication categories based on reported behaviour: (1) MSM who reported anal sex (2) women who reported anal sex and (3) women who did not report anal sex. Demographic data were compared over categories using the student’s T-test and ANOVA. Anorectal load values were compared between indication categories (MSM who reported anal sex, women who reported anal sex and women who did not report anal sex) using the student’s T-test and univariable linear regression analysis using the second category (women who reported anal sex) as the reference category. Univariable and multivariable linear regression analyses were performed to identify determinants associated with anorectal load overall and separately for each indication category. Additional analyses were performed to assess determinants for high load (i.e., the upper quartile versus the other quartiles). Determinants tested were: sex, age, nationality, number of sex partners in the past six months, sexual preference (for MSM), antibiotic use in the past month, symptoms in the past month (anorectal and urogenital), anal sex in the past six months, and concurrent STI (urogenital chlamydia, anorectal gonorrhoea, syphilis (TPHA positivity) and HIV). Analyses were performed using the SPSS package version 20 (IBM Inc. Somers, New York, USA).

## Results

### Characteristics of the study population

Anal sex was reported by 45.5% (51/112) of the women. The median age of the MSM (n = 90) was 36 years (IQR 25–47). It was 25 years (IQR 19–27) for women who reported anal sex (n = 51) and 25 years (IQR 20–26) for women who did not report anal sex (n = 61; p<0.001). The median number of sex partners was 12 (IQR 3–11) for MSM, 4 (IQR 1–4) for women who reported anal sex and 10 (IQR 1–3) for women who did not report anal sex. Anal symptoms were reported by 20.0% (n = 17) of MSM, 15.7% (n = 8) of women who reported anal sex and 3.5% (n = 2) of women who did not report anal sex (p<0.001). In total, 75.8% (n = 72) of MSM in the sample reported only having sex with men and 24.2% (n = 23) reported having sex with both men and women. These and other characteristics are described in Table 1.

**Table 1:** Mean log-transformed number of chlamydia copies per millilitre (Ct/ml) (anorectal load) and associated determinants in MSM, women who reported anal sex and women who did not report anal sex by univariate linear regression analyses.

	MSM who reported anal sex N = 90				Women who reported anal sex N = 51				Women who did not report anal sex N = 61			
	% (N)	Mean log load (SD)	Δ load‡	95% CI	% (N)	Mean log load (SD)	Δ load‡	95% CI	% (N)	Mean log load (SD)	Δ load‡	95% CI
Anorectal load		3.5(1.4)	-0.33	-0.85–0.19		3.8 (1.7)	ref			2.8 (1.6)	-1.04*	-1.67–-0.41
Adjusted anorectal load			0.29	-0.34–0.92			ref				-1.01*	-1.67–-0.035
Age												
16–21	11.1 (10)	3.9 (1.1)	ref		47.1 (24)	4.1 (1.9)	ref		52.2 (32)	2.9 (1.5)	ref	
22–32	32.2 (29)	3.4 (1.6)	-0.42	-1.41–0.57	41.2 (21)	3.6 (1.7)	-0.51	-1.55–0.53	31.1 (19)	2.6 (1.8)	-0.26	-1.22–0.70
33+	56.7 (51)	3.4 (1.2)	-0.43	-1.37–0.50	11.8 (6)	3.6 (1.1)	-0.44	-2.03–1.14	16.4 (10)	2.7 (1.9)	-0.18	-1.38–1.03
Nationality												
Western	94.4 (84)	3.5 (1.3)	ref		98.0 (50)	3.7 (1.7)	na	na	95.0 (57)	2.8 (1.6)	ref	
Non Western	5.6 (5)	3.1 (1.4)	-0.32	-1.55–0.92	2.9 (1)	6.4 (-)	na	na	5.0 (3)	2.0 (1.3)	-0.88	-2.82–1.06
Number of sex partners												
1–2	16.2 (14)	3.6 (1.2)	ref		53.1 (26)	3.5 (1.6)	ref		62.7 (37)	2.8 (1.6)	ref	
3–5	35.6 (31)	3.5 (1.4)	-0.04	-0.91–0.84	30.6 (15)	4.0 (1.8)	0.53	-0.53–1.59	23.7 (14)	2.7 (1.8)	-0.14	-1.19–0.91
6+	48.3 (42)	3.3 (1.4)	-0.27	-1.11–0.57	16.3 (8)	4.6 (1.5)	1.14	-0.19–2.46	13.6 (8)	2.7 (1.9)	-0.19	-1.50–1.11
Antibiotics												
No	86.5 (77)	3.4 (1.4)	ref		86.3 (44)	3.9 (1.7)	ref		87.5 (49)	2.9 (1.6)	ref	
Yes	6.7 (6)	4.3 (0.7)	0.43	-0.10–0.95	13.7 (7)	3.2 (1.5)	-0.74	-2.14–0.66	12.5 (7)	1.9 (1.2)	-0.07	-0.74–0.89
Urogenital symptoms												
No	83.5 (71)	3.5 (1.3)	ref		72.5 (37)	3.7 (1.8)	ref		81.0 (47)	2.8 (1.6)	ref	
Yes	16.5 (14)	3.3 (1.6)	-0.20	-0.99–0.59	27.5 (14)	4.0 (1.6)	0.22	-0.87–1.31	19.0 (11)	2.3 (1.5)	-0.50	-1.59–0.59
Anorectal symptoms												
No	80.0 (68)	3.4 (1.3)	ref		84.3 (43)	3.7 (1.7)	ref		96.5 (55)	2.6 (1.6)	ref	
Yes	20.0 (17)	3.5 (1.4)	0.04	-0.70–0.77	15.7 (8)	4.2 (1.7)	0.47	-0.87–1.80	3.5 (2)	2.8 (0.6)	0.19	-2.13–2.52
Concurrent urogenital chlamydia infection†												
No	86.5 (77)	3.4 (1.4)	ref									
Yes	13.5 (12)	4.0 (1.3)	0.60	-0.23–1.43								
TPHA positive†												
Not tested	65.6 (59)	3.4 (1.3)	-0.41	-0.99–0.16								
No	26.7 (24)	3.7 (1.6)	ref									
Yes	7.8 (7)	3.5 (0.9)	-0.16	-1.45–1.13								
HIV positive†												
No	72.2 (65)	3.4 (1.5)	ref									
Yes	26.7 (24)	3.7 (1.0)	0.37	-0.27–1.02								
Anorectal gonorrhoea†												
No	84.4 (76)	3.5 (1.4)	ref									
Yes	15.6 (14)	3.1 (1.2)	-0.47	-1.25–0.31								

^†^ Concurrent STIs were only assessed as a determinant for MSM since all the women had concurrent urogenital chlamydia and tested negative for TPHA and HIV; all but one woman were also negative for anorectal gonorrhoea.

^‡^ The regression coefficient is represented as Δ load, which represents the change in anorectal chlamydia load between the categories.

Sensitivity analyses were performed by excluding unquantifiable (0.75 log) load samples in analyses, but results remained the same.

Na = not assessed.

* P<0.05.

### Anorectal chlamydial load in women and MSM

**Fig 1:**
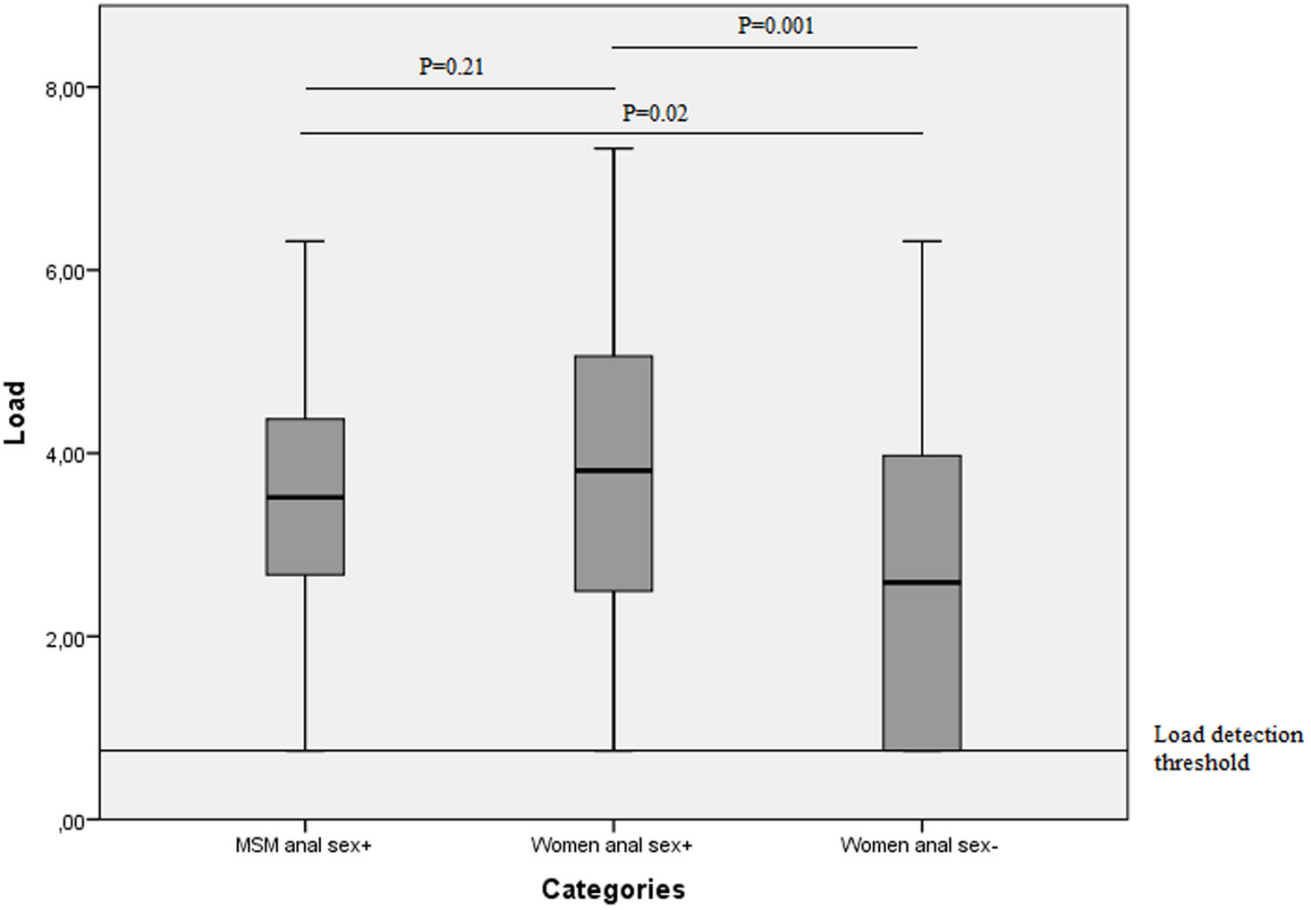
Log-transformed number of cycle threshold per millilitre (Ct/ml) (load) in MSM, women who reported anal sex and women who did not report anal sex, including load detection threshold, geometrical mean and mean difference between groups tested by univariate linear regression analyses.

MSM who reported anal sex had a similar mean log anorectal chlamydia load (3.50) as women who reported anal sex (3.80; Fig 1, Table 1). In multivariable analyses adjusting for age, anal symptoms, urogenital symptoms and number of sex partners, results remained similar (B 0.29, CI -0.34–0.92, P = 0.37). Anorectal chlamydia load was significantly higher in women who reported anal sex (3.80) than in women who did not report anal sex (2.8, P = 0.001). In multivariable analyses, results remained similar (B 1.01, CI 0.35–1.67, P = 0.003). When samples with unquantifiable load were excluded from analyses, results comparing load values between groups remained similar. The proportion of samples with an unquantifiable load was 8.9% (n = 8) for MSM who reported anal sex, 9.8% (n = 5) for women who reported anal sex and 27.9% (n = 17) for women who did not report anal sex (P = 0.03). Detectable load values ranged from 1.81–6.32 chlamydia/ml for MSM, 1.74–7.33 chlamydia/ml for women who reported anal sex and 1.84–6.31 chlamydia/ml for women who did not report anal sex (Fig 1). Among detectable load values, MSM who reported anal sex had a similar mean log anorectal chlamydia load (3.74) as women who reported anal sex (4.13, P = 0.09). Women who did not report anal sex (3.53) had lower mean log anorectal chlamydia load compared to women who did report anal sex (4.14, P = 0.04) among detectable load values.

### Determinants associated with anorectal load

None of the assessed determinants were significantly associated with anorectal load. Although in multivariable analyses adjusted for proctitis and age, antibiotic use in the past month was marginally significantly associated with anorectal chlamydia load. Individuals who used antibiotics in the past month had a 0.91 chlamydia/ml (95% CI -0.01–1.83, P = 0.05) higher anorectal chlamydia load compared to individuals who did not use antibiotics in the past month. None of the assessed determinants were associated with high load (i.e. the upper quartile versus the other quartiles).

## Discussion

Anorectal chlamydial load was comparable between MSM and women who reported anal sex. Women who did not report anal sex had a lower anorectal load, but within a comparable range to the other groups. Having anorectal symptoms was not associated with anorectal load in either MSM or women.

Anorectal chlamydia is known to be equally prevalent in each of the three groups [9,17]. We found an equal load in MSM and women who reported anal sex and no association with symptoms. This may suggest that the direct clinical impact (in terms of symptoms) and the public health impact (in terms of transmission potential) was similar between the two groups. In MSM and women reporting anal sex, chlamydia detection is at least in part caused by transmission due to anal sex. It is likely that transmission by anal sex causes the difference in mean load between women who did and did not report anal sex. Yet, there may be different causes for detection of anorectal chlamydia that affect the potential for further spread.

The anorectal chlamydia load in women who did not report anal sex remains unexplained. In these women, the mean load was lower, yet load values were in the same ranges as for MSM or women who reported anal sex. Moreover, the load differed with log 1 between women who did or did not report anal sex, which is lower than the mean load in women who did not report anal sex (log 2.8). This indicates that transmission routes other than anal sex could lead to substantial anorectal chlamydia load (range 1.84–6.31) in women. Previous studies suggest that autoinoculation (self infection) through vaginal secretions [15,29–31], concurrent transmission during sex [9] or infection via the intestinal canal[32,33] could lead to microbiological detection in women. After oropharyngeal chlamydia infection, the intestinal canal could serve as a reservoir that allows chlamydiae to persist indefinitely by immune down-regulation in the gut[32,33]. However, no studies in humans have confirmed any of these theories. Nevertheless, autoinoculation seems a very likely explanation for the substantial load because of anatomical proximity. Moreover, the majority (up to 95%) of women with an anorectal infection have a concurrent urogenital infection [8,9,18].

In contrast, the majority of MSM with an anorectal infection have a single-site infection [10,12] (i.e. without urogenital infection). Unfortunately MSM who did not report anal sex were not included in this study. It would be interesting to further study whether load differs according to anal sexual behaviour in MSM.

Since the sexual history asked about behaviour in the past six months, the women could have had anal sex with a chlamydia positive partner more than six months prior to consultation. Thereby, the recall period may be too short and the sexual history might not always be trustworthy. Possibly, MSM and women may have incorrectly reported whether or not they have had anal sex.

Contamination during sampling, although unlikely, cannot be completely ruled out. However, contamination by inadequate swab handling is unlikely to play a role since self-collected rectal samples are as valid for testing as provider-collected swabs [3,4]. Environmental contamination is unlikely since laboratory quality procedures reveal that positive samples do not cluster (data not shown). Women who did not report anal sex had a higher proportion of unquantifiable load samples compared to MSM and women who report anal sex. This finding concurs with the overall lower detectable load in this group. It is not surprising that these unquantifiable load samples are tested positive with NAAT, but have a low load below detection level as the quantitative PCR targets a single-copy gene and the NAAT a multi-copy gene. We cannot completely rule out false positives among these unquantifiable load samples. However, this would be very unlikely as routine CE certified NAATs with high sensitivity and specificity were used for diagnostics. Moreover, there is no reason to assume this would be different for MSM and women.

Anal symptoms were not associated with anorectal load for either MSM or women. Other determinants tested (e.g. age, nationality, number of sex partners or concurrent urogenital chlamydia) were not associated with anorectal load in MSM or women. A study by Twin *et al*. also concluded that there were no associations between anorectal chlamydia load and prior chlamydia infection, symptoms or any demographic information [22]. This makes it difficult to target individuals with high load in care based on symptoms [15]. Targeting individuals with a high load based on the report of anal sex may be an option. Yet it has limitations, as half of all anorectal infections in MSM and women would be missed based on previous studies[34]. Moreover, women who did not report anal sex also have high anorectal chlamydia load (up to 6.31). Nevertheless, the extent to which chlamydia load reflects transmission potential or further consequences for morbidity (e.g. reproductive health in women) remains to be determined.

A systematic review of the epidemiology of organism load in genital chlamydia infection by Vodstrcil *et al*. revealed that load varies by specimen type and site of sampling specimens. Culture studies were more likely to have found an association between load and symptoms than NAAT studies [16]. This could indicate that viable organisms, such as those measured by culture, may be more relevant than total load, which is measured by NAAT. It is unknown whether this would be any different between MSM and women. This makes chlamydia viability an important future research area[16].

More research is needed to gain insight into the actual transmission of chlamydia, especially at anorectal sites. Given the lack of better data based on innovative laboratory measurement and transmission studies, and considering the similar load in MSM and women—whatever the reasons for detection may be—we are likely looking at real infections with potential clinical and transmission impacts. State-of-the-art practice acknowledges that anorectal chlamydia in MSM is relevant in terms of testing and treatment. This study shows that anorectal infections in women who report anal sex are of equal relevance, as chlamydia load values are comparable between MSM and women. Anorectal infections are contributing to the growing number of observed chlamydia cases in MSM and, according to recent studies, also in women [8,9,15]. Although the exact impact of load is yet unclear, testing and treatment of anorectal infections seems to remain important to preventing further spread and complications.

This study had several limitations. For practical reasons, a convenience sample was used in this study. The individuals included were, on average, younger than excluded individuals, precluding analyses in older individuals. There is some evidence to suggest that organism load in urogenital samples is higher in young individuals than older individuals[16]. MSM were on average older compared to women, which could have influenced load values. Nevertheless, anorectal chlamydia load was not associated with age in our current study, so we expect bias to be minimal. In addition, current data were limited since women with a single-site anorectal infection and MSM who did not report anal sex were not included in this study. This makes it difficult to generalise results to all MSM and women. Moreover, anal sex in MSM could not be stratified into receptive and insertive anal sex, possibly leading to the misclassification of receptive anal sex. Although the overall number of partners was recorded, there were no data available on the frequency of anal sex among MSM; exposure may have been higher, leading to a possible overestimation of the anorectal load in MSM. More research is needed to compare anorectal chlamydia load in MSM with and without receptive anal sex, and women with and without concurrent infection.

A general limitation encountered in chlamydia load studies is the lack of knowledge about the duration and load curve of a chlamydia infection. Differences in load may be due to different sampling moments during infection[35]. Possibly uncaptured bacterial factors (i.e. chlamydia genotype, local microbiome and co-infections like candida or HPV) or host factors (i.e. local immunity and microbiome) could explain variations in chlamydia load and it is unknown how they affected the findings. Animal models could be useful to obtain more insight in replication dynamics[35], although it is unclear whether these mechanisms are comparable to humans. Samples with a high bacterial load may be presumed to be sampled at the height of infection, while lower loads may indicate the beginning or resolution of a chlamydia infection[28]. Persistent chronic infections, with a low load which remains stable over a long period of time, are also suggested in literature[36,37].

Some recommend standardisation of organism load measures by correcting for the number of human cells present to account for sampling variability[16]. Counterarguments have put forward that commonly used human targets such as HLA-, beta-globin- or beta-actin genes are too broad a target for human cell quantification via PCR. They are present in all cells (e.g. columnar, squamous and inflammatory cells), not just in columnar cells, which is the cell type preferred by chlamydia[38]. It is to question whether normalisation of chlamydia by total human cell count provides relevant information about the chlamydia per epithelial cell concentration[39]. Caution must be used in samples with high inflammatory cell counts, as normalisation will result in low chlamydia/cell loads. This is important in studies correlating clinical features to the chlamydia load, as any positive relation might be masked using such an artificial low load. In this study we did not correct for human cells, i.e. HLA, as we cannot differentiate between leukocytes and epithelial cells with our PCR-assay. It is unlikely that this is a limitation to our study as results remained similar when we corrected for human cells (data not shown).

In conclusion, anorectal chlamydia infections in women who reported anal sex have similar bacterial loads as anorectal infections in MSM. This may imply similar transmission potential and clinical relevance. More research on anorectal chlamydia load is needed (e.g. viability and its role in transmission potential, and the development of sequelae).

## References

[1] John R. Papp JS, Charlotte A. Gaydos, Barbara Van Der Pol. Recommendations for the Laboratory-Based Detection of *Chlamydia trachomatis* and *Neisseria gonorrhoeae*—2014.

[2] Schachter J, Philip SS. Testing men who have sex with men for urethral infection with Chlamydia trachomatis and Neisseria gonorrhoeae is only half the job, and we need the right tools. Sex Transm Dis. 2011;38: 925–927. 10.1097/OLQ.0b013e318230f3d6 21934566

[3] van der Helm JJ, Hoebe CJ, van Rooijen MS, Brouwers EE, Fennema HS, Thiesbrummel HF, et al High performance and acceptability of self-collected rectal swabs for diagnosis of Chlamydia trachomatis and Neisseria gonorrhoeae in men who have sex with men and women. Sex Transm Dis. 2009;36: 493–497. 10.1097/OLQ.0b013e3181a44b8c 19617869

[4] Moncada J, Schachter J, Liska S, Shayevich C, Klausner JD. Evaluation of self-collected glans and rectal swabs from men who have sex with men for detection of Chlamydia trachomatis and Neisseria gonorrhoeae by use of nucleic acid amplification tests. J Clin Microbiol. 2009;47: 1657–1662. 10.1128/JCM.02269-08 19369445 PMC2691064

[5] Workowski KA, Berman SM. Sexually transmitted diseases treatment guidelines, 2006. MMWR Recomm Rep. 2006;55: 1–94. 16888612

[6] 2006 UK National Guideline for the Management of Genital Tract Infection with *Chlamydia trachomatis* Available: http://www.bashh.org/documents/65.pdf

[7] Barry PM, Kent CK, Philip SS, Klausner JD. Results of a program to test women for rectal chlamydia and gonorrhea. Obstet Gynecol. 2010;115: 753–759. 10.1097/AOG.0b013e3181d444f6 20308835

[8] Javanbakht M, Gorbach P, Stirland A, Chien M, Kerndt P, Guerry S. Prevalence and correlates of rectal Chlamydia and gonorrhea among female clients at sexually transmitted disease clinics. Sex Transm Dis. 2012;39: 917–922. 10.1097/OLQ.0b013e31826ae9a2 23191945

[9] van Liere GA, Hoebe CJ, Wolffs PF, Dukers-Muijrers NH. High co-occurrence of anorectal chlamydia with urogenital chlamydia in women visiting an STI clinic revealed by routine universal testing in an observational study; a recommendation towards a better anorectal chlamydia control in women. BMC Infect Dis. 2014;14: 274 10.1186/1471-2334-14-274 24885306 PMC4032161

[10] Kent CK, Chaw JK, Wong W, Liska S, Gibson S, Hubbard G, et al Prevalence of rectal, urethral, and pharyngeal chlamydia and gonorrhea detected in 2 clinical settings among men who have sex with men: San Francisco, California, 2003. Clin Infect Dis. 2005;41: 67–74. 15937765 10.1086/430704

[11] van Liere GA, Hoebe CJ, Dukers-Muijrers NH. Evaluation of the anatomical site distribution of chlamydia and gonorrhoea in men who have sex with men and in high-risk women by routine testing: cross-sectional study revealing missed opportunities for treatment strategies. Sex Transm Infect.90: 58–60. 10.1136/sextrans-2013-051248 24106338

[12] Patton ME, Kidd S, Llata E, Stenger M, Braxton J, Asbel L, et al Extragenital gonorrhea and chlamydia testing and infection among men who have sex with men—STD Surveillance Network, United States, 2010–2012. Clin Infect Dis. 2014;58: 1564–1570. 10.1093/cid/ciu184 24647015 PMC4666527

[13] Cachay ER, Sitapati A, Caperna J, Freeborn K, Lonergan JT, Jocson E, et al Denial of risk behavior does not exclude asymptomatic anorectal sexually transmitted infection in HIV-infected men. PLoS One. 2009;4: e8504 10.1371/journal.pone.0008504 20041143 PMC2794382

[14] Sexual attitudes and lifestyles in Britain: Highlights from Natsal-3 from. Available: http://www.natsal.ac.uk/media/2102/natsal-infographic.pdf.

[15] Ding A, Challenor R. Rectal Chlamydia in heterosexual women: more questions than answers. Int J STD AIDS. 2013;25: 587–592. 24352134 10.1177/0956462413515637

[16] Vodstrcil LA, McIver R, Huston WM, Tabrizi SN, Timms P, Hocking JS. The epidemiology of organism load in genital Chlamydia trachomatis infection—a systematic review. J Infect Dis. 2014;211: 1628–1645. 10.1093/infdis/jiu670 25492913

[17] Peters RP, Dubbink JH, van der Eem L, Verweij SP, Bos ML, Ouburg S, et al Cross-sectional study of genital, rectal, and pharyngeal Chlamydia and gonorrhea in women in rural South Africa. Sex Transm Dis. 2014;41: 564–569. 10.1097/OLQ.0000000000000175 25118973

[18] Bax CJ, Quint KD, Peters RP, Ouburg S, Oostvogel PM, Mutsaers JA, et al Analyses of multiple-site and concurrent Chlamydia trachomatis serovar infections, and serovar tissue tropism for urogenital versus rectal specimens in male and female patients. Sex Transm Infect. 2011;87: 503–507. 10.1136/sti.2010.048173 21856696

[19] Koedijk FD, van Bergen JE, Dukers-Muijrers NH, van Leeuwen AP, Hoebe CJ, van der Sande MA. The value of testing multiple anatomic sites for gonorrhoea and chlamydia in sexually transmitted infection centres in the Netherlands, 2006–2010. Int J STD AIDS. 2012;23: 626–631. 10.1258/ijsa.2012.011378 23033514

[20] Wawer MJ, Gray RH, Sewankambo NK, Serwadda D, Li X, Laeyendecker O, et al Rates of HIV-1 transmission per coital act, by stage of HIV-1 infection, in Rakai, Uganda. J Infect Dis. 2005;191: 1403–1409. 15809897 10.1086/429411

[21] Dukers-Muijrers NH, Speksnijder AG, Morre SA, Wolffs PF, van der Sande MA, Brink AA, et al Detection of anorectal and cervicovaginal Chlamydia trachomatis infections following azithromycin treatment: prospective cohort study with multiple time-sequential measures of rRNA, DNA, quantitative load and symptoms. PLoS One. 2013;8: e81236 10.1371/journal.pone.0081236 24278400 PMC3835673

[22] Twin J, Moore EE, Garland SM, Stevens MP, Fairley CK, Donovan B, et al Chlamydia trachomatis genotypes among men who have sex with men in Australia. Sex Transm Dis. 2011;38: 279–285. 10.1097/OLQ.0b013e3181fc6944 21085058

[23] Linssen CF, Jacobs JA, Stelma FF, van Mook WN, Terporten P, Vink C, et al Herpes simplex virus load in bronchoalveolar lavage fluid is related to poor outcome in critically ill patients. Intensive Care Med. 2008;34: 2202–2209. 10.1007/s00134-008-1231-4 18679655

[24] Vliegen I, Duijvestijn A, Stassen F, Bruggeman C. Murine cytomegalovirus infection directs macrophage differentiation into a pro-inflammatory immune phenotype: implications for atherogenesis. Microbes Infect. 2004;6: 1056–1062. 15380774 10.1016/j.micinf.2004.05.020

[25] Jalal H, Stephen H, Curran MD, Burton J, Bradley M, Carne C. Development and validation of a rotor-gene real-time PCR assay for detection, identification, and quantification of Chlamydia trachomatis in a single reaction. J Clin Microbiol. 2006;44: 206–213. 16390971 10.1128/JCM.44.1.206-213.2006 PMC1351959

[26] van der Helm JJ, Sabajo LO, Grunberg AW, Morre SA, Speksnijder AG, de Vries HJ. Point-of-care test for detection of urogenital chlamydia in women shows low sensitivity. A performance evaluation study in two clinics in Suriname. PLoS One. 2012;7: e32122 10.1371/journal.pone.0032122 22393383 PMC3290553

[27] Sambrook J, Fritsch E, Maniatis T (1989) Molecular cloning: a laboratory manual: Cold Spring Harbor Laboratory Press.

[28] Dirks JA, Wolffs PF, Dukers-Muijrers NH, Brink AA, Speksnijder AG, Hoebe CJ. Chlamydia trachomatis load in population-based screening and STI-clinics: implications for screening policy. PLoS One. 2015;10: e0121433 10.1371/journal.pone.0121433 25826298 PMC4380475

[29] Barry PM, Kent CK, Philip SS, Klausner JD. Results of a program to test women for rectal chlamydia and gonorrhea. Obstet Gynecol. 2010;115: 753–759. 10.1097/AOG.0b013e3181d444f6 20308835

[30] Hunte T, Alcaide M, Castro J. Rectal infections with chlamydia and gonorrhoea in women attending a multiethnic sexually transmitted diseases urban clinic. Int J STD AIDS. 2010;21: 819–822. 10.1258/ijsa.2010.009279 21297090 PMC5440116

[31] Sethupathi M, Blackwell A, Davies H. Rectal Chlamydia trachomatis infection in women. Is it overlooked? Int J STD AIDS. 2010;21: 93–95. 10.1258/ijsa.2008.008406 19917639

[32] Rank RG, Yeruva L. Hidden in plain sight: chlamydial gastrointestinal infection and its relevance to persistence in human genital infection. Infect Immun. 2014;82: 1362–1371. 10.1128/IAI.01244-13 24421044 PMC3993372

[33] Yeruva L, Spencer N, Bowlin AK, Wang Y, Rank RG. Chlamydial infection of the gastrointestinal tract: a reservoir for persistent infection. Pathog Dis. 2013;68: 88–95. 10.1111/2049-632X.12052 23843274 PMC3751173

[34] van Liere GA, Hoebe CJ, Niekamp AM, Koedijk FD, Dukers-Muijrers NH. Standard symptom- and sexual history-based testing misses anorectal Chlamydia trachomatis and neisseria gonorrhoeae infections in swingers and men who have sex with men. Sex Transm Dis. 2013;40: 285–289. 10.1097/OLQ.0b013e31828098f8 23486492

[35] Price MJ, Ades AE, Angelis DD, Welton NJ, Macleod J, Soldan K, et al Mixture-of-exponentials models to explain heterogeneity in studies of the duration of Chlamydia trachomatis infection. Stat Med. 2012;32: 1547–1560. 10.1002/sim.5603 22949217

[36] Caldwell HD, Wood H, Crane D, Bailey R, Jones RB, Mabey D, et al Polymorphisms in Chlamydia trachomatis tryptophan synthase genes differentiate between genital and ocular isolates. J Clin Invest. 2003;111: 1757–1769. 12782678 10.1172/JCI17993 PMC156111

[37] Hogan RJ, Mathews SA, Mukhopadhyay S, Summersgill JT, Timms P. Chlamydial persistence: beyond the biphasic paradigm. Infect Immun. 2004;72: 1843–1855. 15039303 10.1128/IAI.72.4.1843-1855.2004 PMC375192

[38] Moorman DR, Sixbey JW, Wyrick PB. Interaction of Chlamydia trachomatis with human genital epithelium in culture. J Gen Microbiol. 1986;132: 1055–1067. 3760816 10.1099/00221287-132-4-1055

[39] Jalal H, Verlander NQ, Kumar N, Bentley N, Carne C, Sonnex C. Genital chlamydial infection: association between clinical features, organism genotype and load. J Med Microbiol.60: 881–888. 10.1099/jmm.0.028076-0 21415209

